# Supplementary material for: Knowledge and Practices of Four Onchocerciasis-Endemic Communities in Cameroon
Source: Microorganisms. 2025 Mar 25;13(4):736. doi: 10.3390/microorganisms13040736 (PMC12029838; doi:10.3390/microorganisms13040736)
Supplement: Supplementary file 1 [file microorganisms-13-00736-s001.zip › microorganisms-3461677-supplementary.pdf]

Supplementary Table S1. Structured questionnaire

Structured questionnaires prepared based on the socio-cultural history of the community. The participants were interviewed in "Pidjin" a special English dialect mainly used in the Menchum valley and Fulfulde in the Northern part of Cameroon. Respondents, who came near the *Simulium* breeding site where female flies came to bite, were recruiting using systematic sampling.

No………………………Tel…………………………….Date………………………

Gender

☐Male ☐Female

Age

☐Less than 20 ☐30 to 35 ☐45 to 50

☐20 to 25 ☐ 40 to 45 ☐ 50 and over

Education level

☐Primary ☐Secondary ☐Other

Period of the black fly bite

☐Morning ☐Evening ☐Afternoon ☐At any time

Preferred season

☐Dry ☐rainy ☐all seasons

Favourite place

☐River ☐Fields ☐House ☐Office ☐Road ☐Everywhere

Development site

☐Standing water ☐Rapids ☐ Falls ☐Running water ☐Tree hols ☐No idea

Preferred body part

☐Foot/legs ☐Hands ☐The face ☐All exposed part

Effect of the bite

☐Blindness ☐Tuberculosis ☐ Malaria ☐Headache ☐pruritus

☐Asthenia ☐Dermatitis ☐Growth retardation ☐Epilepsy

Your profession ____
